# Supplementary material for: Laboratory Diagnostics of Rickettsia Infections in Denmark 2008–2015
Source: Biology (Basel). 2020 Jun 19;9(6):133. doi: 10.3390/biology9060133 (PMC7345066; doi:10.3390/biology9060133)
Supplement: Supplementary file 1 [file biology-09-00133-s001.pdf]

biology

MDPI

Partial 16S RNA sequences obtained from samples that tested positive for *Rickettsia* gltA by real-time PCR.

>#01
cttaacacatgcaagtcgaacgaactaatttttggggcttgctccaattagttagtggcagacgggtgagtaacacgtgggaatctacccattagt
acggaataacttttagaaataaaagctaataccgtatattctctgcggaggaaagatttatcgctgatggatgagcccgcgtcagattaggtagtt
ggtgaggtaatggctcaccaagccgacgatctgtagctggtctgagaggatgatcagccacactgggactgagacacggcccagactcctacg
ggaggcagcagtggggaatattggacaatgggcgaaagcctgatccagcaataccgagtgagtgatgaaggccttagggttgtaaagctcttt
tagcaaggaagataatgacgttacttgcagaaaaagccccggctaactccgtgccagcagccgcggtaagacggagggggctagcgttgttcg
gaattactgggcgtaaagagtgcgtaggc
>#02
tagttagtggcagacgggtgagtaacacgtgggaatctacccattagtacggaataacttttagaaataaaagctaataccgtatattctctgcgg
aggaaagatttatcgctgatggatgagcccgcgtcagattaggtagttggtgaggtaatggctcaccaagccgacgatctgtagctggtctgag
aggatgatcagccacactgggactgagacacggcccagactcctacgggaggcagcagtggggaatattggacaatgggcgaaagcctgat
ccagcaataccgagtgagtgatgaaggccttagggttgtaaagctcttttagcaaggaagataatgacgttacttgcagaaaaagccccggcta
actccgtgccagcagccgcggtaagacggagggggctagcgttgttcggaattactgggcgtaaagagtgcgtaggcggtttagtaagt
>#03
gctatcggtatgcttaacacatgcaagtcgaacgaactaatttttggggcttgctccaattagttagtggcagacgggtgagtaacacgtgggaat
ctacccattagtacggaataacttttagaaataaaagctaataccgtatattctctgcggaggaaagatttatcgctgatggatgagcccgcgtcag
attaggtagttggtgaggtaatggctcaccaagccgacgatctgtagctggtctgagaggatgatcagccacactgggactgagacacggccca
gactcctacgggaggcagcagtggggaatattggacaatgggcgaaagcctgatccagcaataccgagtgagtgatgaaggccttagggttg
taaagctcttttagcaaggaagataatgacgttacttgcagaaaaagccccggctaactccgtgccagcagccgcggtaagacggagggggct
agcgttgttcggaattactgggcgtaaagagtgcgtaggcggtttagtaagtt
>#04
ctacgccactctttacgcccagtaattccgaacaacgctagcccccctccgtcttaccgcggctgctggcacggagttagccggggctttttctgcaa
gtaacgtcattatcttccttgctaaaagagctttacaaccctaaggccttcatcactcactcggtattgctggatcaggctttcgcccattgtccaatatt
ccccactgctgcctcccgtaggagtctgggccgtgtctcagtcccagtgtggctgatcatcctctcagaccagctacagatcgtcggcttggtgagc
cattacctcaccaactacctaatctgacgcgggctcatccatcagcgataaatctttcctccgcagagaatatacggtattagcttttatttctaaaagt
tattccgtactaatgggtagattcccacgtgttactcacccgtctgccactaactaattggagcaagccccaaaaattagttcgttcgacttgcatgtg
ttaa
>#05
gcttaacacatgcaagtcgaacgaactaatttttggggcttgctccaattagttagtggcagacgggtgagtaacacgtgggaatctacccattag
tacggaataacttttagaaataaaagctaataccgtatattctctgcggaggaaagatttatcgctgatggatgagcccgcgtcagattaggtagtt
ggtgaggtaatggctcaccaagccgacgatctgtagctggtctgagaggatgatcagccacactgggactgagacacggcccagactcctacg
ggaggcagcagtggggaatattggacaatgggcgaaagcctgatccagcaataccgagtgagtgatgaaggccttagggttgtaaagctcttt
tagcaaggaagataatgacgttacttgcagaaaaagccccggctaactccgtgccagcagccgcggtaagacggagggggctagcgttgttcg
gaattactgggcgtaaagagtgcgtagg
>#06
gcttaacacatgcaagtcgaacgaactaatttttggggcttgctccaattagttagtggcagacgggtgagtaacacgtgggaatctacccattag
tacggaataacttttagaaataaaagctaataccgtatattctctgcggaggaaagatttatcgctgatggatgagcccgcgtcagattaggtagtt
ggtgaggtaatggctcaccaagccgacgatctgtagctggtctgagaggatgatcagccacactgggactgagacacggcccagactcctacg
ggaggcagcagtggggaatattggacaatgggcgaaagcctgatccagcaataccgagtgagtgatgaaggccttagggttgtaaagctcttt
tagcaaggaagataatgacgttacttgcagaaaaagccccggctaactccgtgccagcagccgcggtaagacggagggggctagcgttgttcg
gaattactgggcgtaaagagtgcgtagg
>#07
cttaacacatgcaagtcgaacgaactaatttttggggcttgctccaattagttagtggcagacgggtgagtaacacgtgggaatctacccattagt
acggaataacttttagaaataaaagctaataccgtatattctctgcggaggaaagatttatcgctgatggatgagcccgcgtcagattaggtagtt
ggtgaggtaatggctcaccaagccgacgatctgtagctggtctgagaggatgatcagccacactgggactgagacacggcccagactcctacg
ggaggcagcagtggggaatattggacaatgggcgaaagcctgatccagcaataccgagtgagtgatgaaggccttagggttgtaaagctcttt
tagcaaggaagataatgacgttacttgcagaaaaagccccggctaactccgtgccagcagccgcggtaagacggagggggctagcgttgttcg
gaattactgggcgtaaagagtgcgtaggcg

>#08
atgcttaacacatgcaagtcgaacgaactaatttttggggcttgctccaattagttagtggcagacgggtgagtaacacgtgggaatctacccatt
agtacggaataacttttagaaataaaagctaataccgtatattctctgcggaggaaagatttatcgctgatggatgagcccgcgtcagattaggta
gttggtgaggtaatggctcaccaagccgacgatctgtagctggtctgagaggatgatcagccacactgggactgagacacggcccagactccta
cgggaggcagcagtggggaatattggacaatgggcgaaagcctgatccagcaataccgagtgagtgatgaaggccttagggttgtaaagctc
ttttagcaaggaagataatgacgttacttgcagaaaaagccccggctaactccgtgccagcagccgcggtaagacggagggggctagcgttgtt
cggaattactgggcgtaaagagtgcgtaggcgg
>#09
cggtatgcttaacacatgcaagtcgaacgaactaatttttggggcttgctccaattagttagtggcagacgggtgagtaacacgtgggaatctacc
cattagtacggaataacttttagaaataaaagctaataccgtatattctctgcggaggaaagatttatcgctgatggatgagcccgcgtcagattag
gtagttggtgaggtaatggctcaccaagccgacgatctgtagctggtctgagaggatgatcagccacactgggactgagacacggcccagact
cctacgggaggcagcagtggggaatattggacaatgggcgaaagcctgatccagcaataccgagtgagtgatgaaggccttagggttgtaaa
gctcttttagcaaggaagataatgacgttacttgcagaaaaagccccggctaactccgtgccagcagccgcggtaagacggagggggctagcg
ttgttcggaattactgggcgtaaagagtgcgtaggcgg
>#10
ttaacacatgcaagtcgaacgaactaatttttggggcttgctccaattagttagtggcagacgggtgagtaacacgtgggaatctacccattagta
cggaataacttttagaaataaaagctaataccgtatattctctgcggaggaaagatttatcgctgatggatgagcccgcgtcagattaggtagttg
gtgaggtaatggctcaccaagccgacgatctgtagctggtctgagaggatgatcagccacactgggactgagacacggcccagactcctacgg
gaggcagcagtggggaatattggacaatgggcgaaagcctgatccagcaataccgagtgagtgatgaaggccttagggttgtaaagctctttt
agcaaggaagataatgacgttacttgcagaaaaagccccggctaactccgtgccagcagccgcggtaagacggagggggctagcgttgttcg
gaattactgggcgtaaagagtgcgtagg
>#11
cttaacacatgcaagtcgaacgaactaatttttggggcttgctccaattagttagtggcagacgggtgagtaacacgtgggaatctacccattagt
acggaataacttttagaaataaaagctaataccgtatattctctgcggaggaaagatttatcgctgatggatgagcccgcgtcagattaggtagtt
ggtgaggtaatggctcaccaagccgacgatctgtagctggtctgagaggatgatcagccacactgggactgagacacggcccagactcctacg
ggaggcagcagtggggaatattggacaatgggcgaaagcctgatccagcaataccgagtgagtgatgaaggccttagggttgtaaagctcttt
tagcaaggaagataatgacgttacttgcagaaaaagccccggctaactccgtgccagcagccgcggtaagacggagggggctagcgttgttcg
gaattactgggcgtaaagagtgcgtaggcggtttagtaagtt
>#12
tagttagtggcagacgggtgagtaacacgtgggaatctacccattagtacggaataacttttagaaataaaagctaataccgtatattctctgcgg
aggaaagatttatcgctgatggatgagcccgcgtcagattaggtagttggtgaggtaatggctcaccaagccgacgatctgtagctggtctgag
aggatgatcagccacactgggactgagacacggcccagactcctacgggaggcagcagtggggaatattggacaatgggcgaaagcctgat
ccagcaataccgagtgagtgatgaaggccttagggttgtaaagctcttttagcaaggaagataatgacgttacttgcagaaaaagccccggcta
actccgtgccagcagccgcggtaagacggagggggctagcgt
>#13
gcttaacacatgcaagtcgaacgaactaatttttggggcttgctccaattagttagtggcagacgggtgagtaacacgtgggaatctacccattag
tacggaataacttttagaaataaaagctaataccgtatattctctgcggaggaaagatttatcgctgatggatgagcccgcgtcagattaggtagtt
ggtgaggtaatggctcaccaagccgacgatctgtagctggtctgagaggatgatcagccacactgggactgagacacggcccagactcctacg
ggaggcagcagtggggaatattggacaatgggcgaaagcctgatccagcaataccgagtgagtgatgaaggccttagggttgtaaagctcttt
tagcaaggaagataatgacgttacttgcagaaaaagccccggctaactccgtgccagcagccgcggtaagacggagggggctagcgttgttcg
gaattactgggcgtaaagagtgcgtagg
